# Evaluation of wastewater surveillance for SARS-CoV-2 in a prison population: a mixed-methods approach

**DOI:** 10.3389/fpubh.2024.1462186

**Published:** 2024-11-19

**Authors:** Gethin Jones, Andrew Nelson, David R. Chadwick, Steve Cobley, Davey L. Jones, Stephanie Perrett, William Bernard Perry, Andrew J. Weightman, Rachel C. Williams, Daniel Rhys Thomas

**Affiliations:** ^1^Communicable Disease Surveillance Centre, Public Health Wales, Cardiff, United Kingdom; ^2^School of Environmental and Natural Sciences, Bangor University, Bangor, United Kingdom; ^3^Science Evidence and Advice Division, Welsh Government, Cardiff, United Kingdom; ^4^Communicable Disease Inclusion Health Programme, Public Health Wales, Cardiff, United Kingdom; ^5^School of Biosciences, Cardiff University, Cardiff, United Kingdom

**Keywords:** surveillance evaluation, wastewater, monitoring, prisons, mixed-methods, COVID-19, SARS-CoV-2

## Abstract

**Background:**

Prisons are high-risk settings for the transmission of communicable disease. Robust surveillance systems are required to identify and control outbreaks. Wastewater surveillance for SARS-CoV-2 was introduced in four prisons in Wales in March 2022. We investigated its contribution to the COVID-19 surveillance programme.

**Methods:**

We evaluated prison wastewater surveillance against eight system attributes using a mixed-methods approach. Semi-structured interviews were completed with key stakeholders to assess usefulness, flexibility and acceptability. Quantitative analyses were completed to assess data quality, sensitivity, positive-predictive value, representativeness and timeliness. To assess sensitivity of the system to detect changes in incidence we carried out a time-series analysis comparing levels of virus in wastewater with trends in confirmed COVID-19 cases from clinical surveillance.

**Results:**

Interviews with stakeholders indicated that wastewater surveillance is a useful adjunct to existing case-based surveillance. However, it had limited influence on action taken within the prison, often lagging behind existing surveillance and not specific enough to target interventions. The novelty of wastewater surveillance meant stakeholders lacked confidence in interpreting the data. Despite these limitations, wastewater surveillance detected changes in SARS-CoV-2 activity in Welsh prison populations which corroborated trends in case surveillance.

**Conclusion:**

Prison wastewater surveillance, implemented in Wales for a period during the COVID-19 pandemic, was useful and should be considered as part of a wider surveillance programme in response to future SARS-CoV-2 waves, or in response to future pandemics. It is particularly beneficial in the absence of comprehensive clinical testing. We identified several limitations to address should this surveillance be re-started.

## Background

Prisons are high-risk settings for transmission of infectious diseases (1), including respiratory viruses such as SARS-CoV-2 (2, 3), with higher reported levels of infection compared to the general population (4). They present a unique challenge for infection control (5) as they are dynamic and densely populated, with inmates held in close confinement with limited fresh-air flow (3, 6) wherein a single imported case is capable of leading to a large-scale outbreak (7). Their populations also comprise vulnerable individuals, including those with underlying physical and mental health conditions, as well as other comorbidities (3, 6, 8), thus increasing risk of severe outcomes following COVID-19 infection (7, 9). Therefore, in addition to robust infection prevention and control (IPC) measures, good surveillance systems are required to identify and track the spread of COVID-19 within the prison environment, helping to identify and control outbreaks.

Wastewater surveillance is a novel approach for the surveillance of SARS-CoV-2 and alongside the Welsh national wastewater monitoring program (10), was introduced in four of six prisons in Wales in March 2022, with formal surveillance reports sent to stakeholders in the period 17th May 2022 and 4th April 2023. Sites were chosen by the Ministry of Justice with no input from Welsh Government or the academic partners involved in the surveillance program. Individuals infected with SARS-CoV-2 shed its associated RNA gene fragments in their feces and occasionally urine (11, 12), regardless of whether they are symptomatic (13, 14). RNA fragments may also be orally shed into the wastewater system via teeth brushing (15). Rates of SARS-CoV-2 can be monitored via the systematic collection of wastewater samples from sewage networks. This provides a representative snapshot of SARS-CoV-2 infection levels within a specific community, with the potential to capture the presence of infected individuals irrespective of symptoms and the prevailing clinical testing policy and practice (16) and with the additional capability of assessing the presence of mutations (14, 17).

Prison populations offer a unique opportunity to study the relationship between wastewater signals and cases reported via clinical testing for SARS-CoV-2 given their relatively static populations and the fact that routine testing in this population continued beyond that of the general population in the UK. We investigate whether this prison wastewater surveillance program was a useful addition to the COVID-19 surveillance program, which relies on clinical testing, and specifically whether it met its objectives to inform the prevention and control of SARS-CoV-2 in prisons.

## Methods

### Aims and objectives

The primary aim of this paper was to evaluate the prison wastewater surveillance program in Wales to establish if it contributes to the prevention and control of adverse events from SARS-CoV-2.

The specific objectives were to:

- Provide an overview of the wastewater surveillance system within the Welsh prison estate in its current form, how it operates and the data it presents.
- Evaluate the usefulness, flexibility, acceptability, data quality, sensitivity, positive-predictive value, representativeness, and timeliness of the prison wastewater surveillance program using a mixed-methods approach with definitions adapted from ECDC and CDC guidelines (18, 19).
- Discuss any identified limitations of the wastewater surveillance program and their implications for the prevention and control of SARS-CoV-2 within the Welsh prison estate.
- Outline recommendations for improvements to the system to improve its utility as a near-source surveillance system.

A mixed-methods approach (20) was adopted for this study to provide a detailed picture of the surveillance system, its purpose, intended use, and how it influences action on the ground, using perspectives of stakeholders with varied priorities and roles within the surveillance system. These perspectives were complimented by a quantitative assessment of the relationship between levels of SARS-CoV-2 in wastewater and clinically confirmed cases.

### Description of system

First, the wastewater surveillance system was described using information provided within distributed reports and supplemented through interviews with stakeholders involved in analysing the data and overseeing the surveillance system.

### Evaluation of system attributes

Once described, the prison wastewater surveillance system was evaluated against eight key system attributes: usefulness, flexibility, acceptability, data quality, sensitivity, positive-predictive value, representativeness and timeliness (18, 19) using a mixed-methods approach.

### Qualitative evaluation

Ten semi-structured interviews were completed between November and December 2022 with 13 key stakeholders selected for their knowledge and experience in either producing, overseeing or acting upon the wastewater surveillance. These included staff from: Welsh Government, Cardiff University School of Biomedical Science, HM Prison and Probation Service (HMPPS), Consultants in Communicable Disease Control (CCDC’s), and COVID-19 Single Point of Contacts (SPOC)^1^ for prisons and prison governors.

Interviews were conducted via Microsoft Teams and ranged between 20 and 60 min, with an average length of 40 min. While most interviews consisted of one participant, two of the interviews involved two participants and three participants, respectively. Interviews were recorded and transcribed. Thematic analysis (21) was carried out to highlight the key themes from the conversations (Supplementary material 1).

Two similar but distinct interview schedules (Supplementary material 2) were developed for use with either those receiving the wastewater reports or those involved in producing them, covering many of the attributes considered in this evaluation.

For those receiving the weekly prison wastewater surveillance reports, the interview focused on three attributes: timeliness, usefulness and acceptability of the reported information. The interviews aimed to consider whether the reports were received regularly and on time, whether participants understood the data in the reports, how useful they found the reports and whether the reports had any impact on decision making. Interviews also included questions regarding suggested improvements to the surveillance report and whether the implementation of the wastewater surveillance had placed any additional burden on them in terms of time and resources.

For those involved in producing the wastewater surveillance reports, the interview focused on four attributes: usefulness, flexibility, data quality and representativeness. These interviews considered how data in the reports should be interpreted, any limitations associated with the data and how the data should be acted upon; whether the surveillance system was flexible enough to detect unknown variants and infections, and how easily additional prisons could be added to the system. Also considered were the types of factors that may influence poor quality samples; whether the data in the reports were representative of the prison population and whether the sampler location may bias results toward specific areas of the prison. A reflective section was also added to the latter interview schedule, covering what participants had felt they had learned from this novel surveillance system and whether it had contributed to any improved understanding of community wastewater surveillance.

### Quantitative evaluation

**Figure 1:**
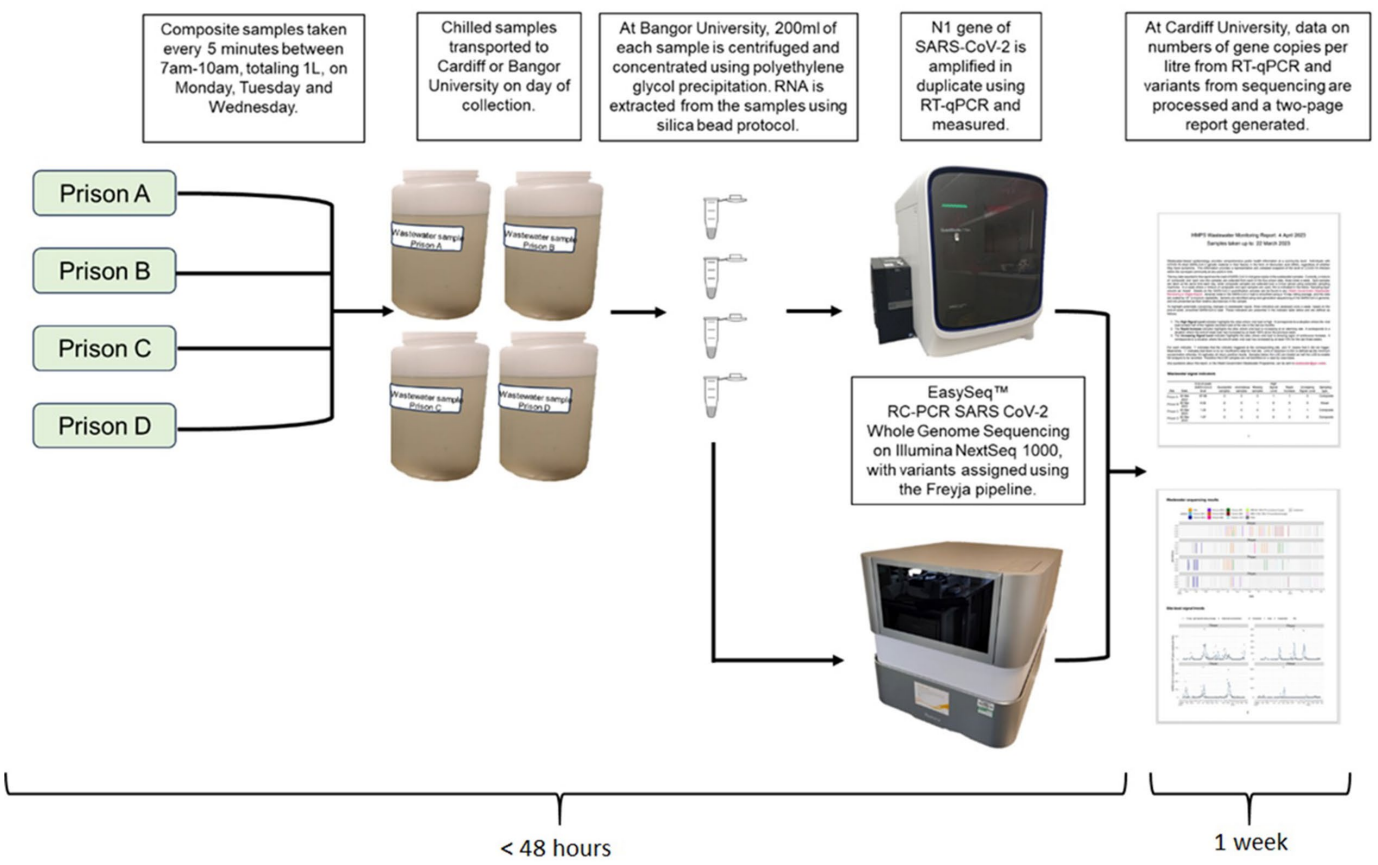
Wastewater processing pipeline from sample collection to report generation.

The quantitative evaluation focused on data quality, timeliness, sensitivity and positive-predictive value (Figure 1).

Reports published between 17th May 2022 and 29th December 2022 were reviewed to evaluate data quality, timeliness and sensitivity. To assess data quality, previous reports were reviewed to determine the number of missing samples and sample failure rate. Timeliness was evaluated by: (1) calculating the average lag time between sample collection and report dissemination, and (2) regularity of report publication.

In the published reports, three indicators were presented to identify significant changes in the wastewater signal:

- High-level Signal indicator: viral load exceeds half of the highest recorded load at the site in the last 6 months.
- Rapid Increase indicator: sites where end-of-week load has increased by at least 100% since the previous week.
- Increasing Trend indicator: sites where viral load is showing signs of continuous increase, i.e., the end-of-week viral load has increased by at least 10% for the last 3 weeks.

Our definition of sensitivity was amended from CDC guidelines (19) for the purpose of event-based surveillance to be the ability to monitor signal changes over time and was evaluated by counting the number of triggers of high-level, rapid increase and increasing trend signals.

Similarly to a previous closed-setting wastewater surveillance evaluation (22) we evaluated positive-predictive value and determined the relationship between wastewater levels and case data by comparing weekly mean wastewater levels (mean gene copies per liter) with the total number of confirmed cases of COVID-19 in each prison between 4^th^ March 2022 and 24th October 2022 using existing clinical surveillance. Wastewater was collected three times a week using refrigerated composite autosamplers and samples quantified using RT-qPCR (23). The time from sample collection to the generation of quantitative data of SARS-CoV-2 levels in wastewater was rapid (<48 h), however, the data were collated to produce a weekly report which was subsequently given to prison staff 1 week later.

To measure the strength and direction of any linear relationship between SARS-CoV-2 gene copies and confirmed SARS-CoV-2 cases, we calculated Pearson’s correlation coefficients. The analysis used mean weekly SARS-CoV-2 gene copies per liter and the total number of confirmed SARS-CoV-2 cases in the same week. The analysis was collapsed across prison. Case data were obtained from existing Public Health Wales surveillance systems, which captured any resident or staff member that tested positive for SARS-CoV-2 by either a PCR or rapid antigen test. Symptomatic testing was available throughout the study period in each of the prisons included, with asymptomatic testing available until 8^th^ September 2022 (although it may have been introduced in response to local risk and outbreak management). Note denominator data were not available. Wastewater samples that did not meet the limit of detection threshold (less than 613.3 gene copies per liter) were excluded from the analysis. The alpha level was set at *p* < 0.05. All analysis was performed in using R studio (version 4.1.3) (24).

## Results

### Description of the system

**Figure 2:**
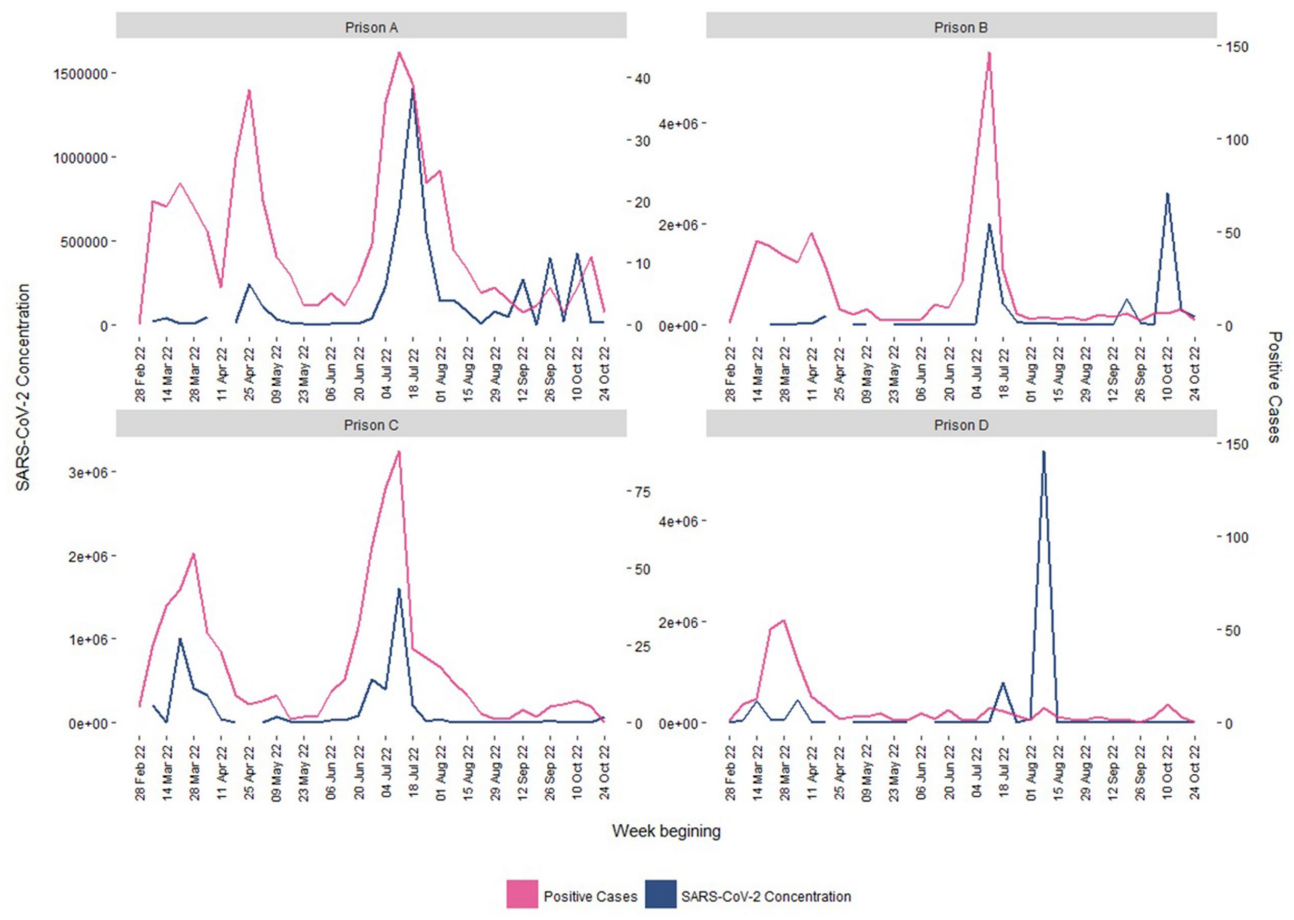
Confirmed positive COVID-19 cases (pink) and average SARS-CoV-2 wastewater concentration levels (gene copies per liter; blue) by week, 04/03/2022–24/10/2022.

Wastewater surveillance was near-sourced, with samples taken just before the main sewer discharge point at the site with samples collected using composite autosamplers. This captured wastewater containing SARS-CoV-2 virus excreted by staff and residents. The autosamplers collected samples every 5 min between 7 am and 10 am on Monday, Tuesday and Wednesday. If automatic sample collection failed, then a grab sample was taken instead at time of collection. Grab samples were collected at the same time each day. Samples were transported at 4°C to laboratories in Cardiff and Bangor University on the day of collection, and all samples were analyzed at Bangor University. Samples were then prepped for the RT-qPCR process to quantify the load of SARS-CoV-2 RNA present in the sample and the number of gene copies per liter are calculated (Figure 2).

### Evaluation of system attributes

The results for each of the attributes evaluated are discussed below and a summary table is available in Supplementary Table S5.

### Usefulness

Stakeholders reported wastewater surveillance was a useful adjunct to existing surveillance, providing intelligence of COVID-19 levels within the prison estate. However, participants noted that it provided a ‘lagging’ rather than a ‘leading’ indicator, limiting usefulness:

“*As the data can be historic, it downgrades its usefulness and importance if it’s 2 weeks old what happened then is probably not as bad now.*” (Governor)

The inability to distinguish between areas of the prison, or between staff and resident cases was also considered to limit the targeting of interventions:

“*It’s more a reflective report, we are reflecting on what’s been, it does not give me enough information to push testing. So more looking at what’s happened, not what’s coming; it’s not useful for prevention. If the data was more active we could be more proactive, we could address it.*” (Governor)

Stakeholders did note that while the wastewater data were not specific enough to target interventions, they could be used to prompt discussions on the ground and increase vigilance among staff. Its primary usefulness, however, was felt to lie in its ability to provide reassurance, particularly during periods of reduced testing capacity, that interventions were effective, and to contribute to the decision to close down outbreak investigations:

“*I think they have helped me when to close things down, it’s reassuring; when the wastewater is not finding anything, we can step down from our meetings now. So it probably has influenced that but nothing else really.*” (CCDC)

The report was used to corroborate other intelligence, but not something that could be relied upon in isolation for decision making:

“*I think it’s useful as an adjunct and another bit of data, it’s providing another bit to the jigsaw but it’s not something I would probably act on in isolation.*” (CCDC)

“*When we had outbreaks the report was used to confirm the judgement, it is used but more as secondary data rather than the main driver in our decision-making.*” (HMPPS Partner)

Government officials reported that wastewater surveillance was intended to be used in conjunction with conventional case surveillance to prompt discussion among colleagues and begin investigations, indicating the wastewater report was being used as intended by participants:

*“It’s a good basis to start discussions to see what’s going on in the prison, and looking at the data for the site cases [from existing surveillance] in combination with the data that we provide. Wastewater, we do not use it as a standalone surveillance, and we never have in Wales.”* (Welsh Government Official)

One complicating factor is that interpretation of the data is more challenging within prisons with fluctuating populations. This adds a layer of complexity to interpretation for remand prisons, given that prisoners may only be housed within these prisons for a short amount of time and have a consistent churn in their housed population:

*“We [academic partner] do not know the size of the prison populations. That information comes back to how you interpret and the actions you take on the results. If you have prison numbers that change a lot that’s going to make interpretation and any actions you take more difficult.”* (Academic Partner)

Given the relative novelty of wastewater as a surveillance system, understanding the information presented in reports varied across stakeholders, with those less accustomed to working with data and statistics wary of interpretation:

“*I have my way of reading it however, I am not a scientist or someone who is trained in any of these things… so having someone who is more knowledgeable and that is their background, some commentary from them would be better in case I’m wrong.*” (HMPPS Partner)

Those without scientific backgrounds relied upon commentary provided by Public Health Wales for interpretation of the data, as they did not feel confident interpreting the reports themselves. The novelty of the surveillance system also meant that the limitations of wastewater surveillance were not well understood by stakeholders:

“*I understand what I suspected should be the limitations but it’s never been explained, so I could be wrong.”* (CCDC)

This affected the confidence stakeholders had in their interpretation of the report, as many had outstanding questions, including: are spikes in wastewater the result of many cases with low viral load, or fewer cases with a higher viral load? What affect does vaccination have on levels of COVID-19 identified via wastewater surveillance? Does the wastewater capture a representative picture of the site? What impact might laundry services have on the signal? However, some stakeholders felt that overburdening the report with notes on interpretation of wastewater surveillance would confuse and limit the usefulness of the message and ability to react, and felt like it was important to keep the report concise and readable.

Academic partners involved in data analysis also expressed frustration that the potential of the prison wastewater surveillance was not realized due to lack of feedback from stakeholders and lack of access to other sources of surveillance data:

“Because w*e do not have any of the metadata surrounding the SARS data, it’s very difficult to interpret exactly what it means;* i.e. *because we do not have case data, because we do not have population data, we do not know where the samplers are, this all limits our ability to interpret what the results mean. And because we have not had any feedback, we have no way of judging whether the data have been useful to the stakeholders … There would be potential for the HMPPS project to be more informative for everyone involved but because the data are not shared and because we have no feedback, it limits the benefits.*” (Academic Partner)

Surveillance of SARS-CoV-2 variants was perceived as the least useful element of the system. For stakeholders less familiar with genomic epidemiology, the data, provided without interpretation in the reports, gave little insight into the impact that the distribution of circulating variants might have on the ground. Stakeholders from the prison instead relied on colleagues from Public Health Wales (PHW) to interpret and flag any concern. For stakeholders with expert knowledge in the field, there was a mix of opinion on the usefulness of the variant surveillance but most tended to rely on other sources for information on circulating variants. While some found it useful data to triangulate with other sources, others were skeptical about its validity:

*“I do not think I would rely on the wastewater [for sequencing information] or pay much heed to it … the wastewater is less reliable as you are looking at scraps of genetic material. There’s a little bit of a concern as to how accurate it is…”* (CCDC)

### Flexibility

The wastewater surveillance system was described as flexible and capable of accurately detecting both existing and emerging SARS-CoV-2 variants, as well as being adaptable for surveillance of alternative infections, such as influenza and norovirus:

*“Yes, the idea of doing sequencing instead of targeted PCR is so we can see* var*iants and new variants as they emerge. Usually the variant has already been identified somewhere in the world, the data is uploaded to various systems. The system can also be adapted for surveillance of other infections like the flu and norovirus.”* (Academic Partner)

This suggests that wastewater surveillance may provide an alternative source of surveillance data for other infections and a potentially useful source of information if sequencing information from other sources is reduced.

The Ministry of Justice were responsible for sampling design, with no input from Welsh Government or academic partners. Conversations with Welsh Government suggest that additional prisons could be added to the system with relative ease, equipment can be easily fitted within new prisons, the only barrier being costs and logistics of installation, sample collection and analysis.

### Acceptability

All stakeholders confirmed that the introduction of the surveillance placed no additional burden on them in terms of time or resource. The surveillance report (Supplementary material 3) was appreciated for being short and concise, and while participants did not always know what to do with the information provided, almost all participants engaged with it.

Whilst no stakeholders had actively sought to give feedback on the report, many felt like they had an avenue to do so, either through PHW or HMPPS colleagues. During the interview process, many raised aspects of the report that they believed could be improved. The table included in the report (Supplementary material 3), which denoted the triggered signal indicators alongside missing and successful samples and the type of sample taken (composite/grab) was considered to be unintuitive and confusing by many. Participants felt the numbering system (0, 1, −1) was difficult to understand, and was further complicated by a lack of understanding of what implications missing samples may have on the data.

As a result of the difficulty stakeholders had interpreting the aforementioned table, HMPPS distributed an altered version (Supplementary material 4) to Governors which provided a more visual presentation of the data, using green and red arrows to designate positive and negative changes in SARS-CoV-2 levels in the wastewater week-on-week, and red ticks to highlight when one of the signals had been triggered. Participants who received this adapted version felt this improved their ability to interpret the data and the speed and clarity in which they could digest the information:

“*[I] receive [the HMPPS] report that they change slightly for [Prison D]. I do prefer that one with the colors as it’s simpler. At a glance, it’s nice and easy. The normal table in the report is more difficult, I usually just go to the bottom [for the graphs].”* (CCDC)

However, this adaptation of the report did impact on its timeliness and occasionally resulted in delays in distribution due to the additional time taken to amend the report to improve accessibility.

The ‘Rapid Increase’ signal indicator presented in the table was also highlighted as potentially misleading given its reliance on the previous weeks data, particularly for those with less familiarity with data and statistics. For example, the doubling of a low level of viral load and a large viral load would both be represented as an increase of 100% and trigger the Rapid Increase indicator, but the latter would be far more concerning:

*“The problem is as it’s making a comparison to the previous report, if you have a very low signal, 100% of a low signal is not a lot but 100% of a high signal is a lot, so it could be misleading. One you are not really bothered about as 100% of one would be two cases which is not bad but 100% of 300 is 600. They are both 100% but you are only bothered about one. This is why I think the pictorial representation is more useful.”* (CCDC)

### Data quality

Over the 31-week period between 17th May and 29th December 2022, there were 213 samples successfully taken from the prison sites, processed in the laboratory through to quantification, yielding a measurable SARS-CoV-2 value, i.e., above the limit of detection threshold. There were a total of 22 (10.3%) missing samples (sample could not be retrieved from the site) across the four participating prisons. Missing samples did not occur with any regularity but were often aggregated. Over the same period, there were more instances of no (or insufficient) data for signals to trigger than positive signal triggers.

There are a number of external factors that contribute to poor quality or missing samples, such as “ragging,” where the sewage system becomes blocked by foreign objects flushed down the toilet:

*“They do sample collections at a particular time between 07:00 am and 10:00 am, which is not a true reflection of the whole day. It’s only a small time-frame that they take those samples from … on the prison environment itself, if inmates put things down the toilet then obviously that’s going to affect the collection for that specific day, and some days it’s completely dry so you cannot even take a grab sample. So given the environment it’s in, it’s always going to be affected by outside influences that we have no control of.”* (Welsh Government Official)

The relatively short window for sample collection is highlighted above as a potential problem for representativeness of the surveillance given that it only covers a relatively brief period in the morning, meaning that it is not a true reflection of levels of SARS-CoV-2 throughout the day. Similarly, if the hardware fails because of, for example, a ragging issue, grab samples are taken, which can provide an unrepresentative account of SARS-CoV-2 levels in the wastewater for that day:

*“They take grab samples if the hardware does not collect, which means you are taking a sample at that particular time so it’s not a great way of doing it but it’s what we follow from the main wastewater surveillance. As it’s [the grab sample] taken at that specific time [when they go to collect the samples], there may be nothing in the wastewater at that particular time when they are taking the grab sample.”* (Welsh Government Official)

It was noted in the interview that grab samples became less frequent as the surveillance system progressed and teething problems were ironed out, but that the requirement for battery-operated samplers due to their location within the prison also caused early issues due to battery packs failing on occasion.

The proximity of the sampler to laundry services within the prison can also degrade the signal:

*“In an internal wastewater system, anything that goes down the sink and into the drains is going to dilute the signal to some extent and we can account for that. But if there’s a laundry nearby the sampling point and it’s pushing out a lot of hot, soapy water for example, or water that is contaminated by other chemicals, that certainly could affect the signal.”* (Academic Partner)

It is possible to account for the dilution caused by those chemicals but there was some frustration among academic partners that they were directed not to undertake any additional chemistry on the samples^2^ to identify those elements that might dilute the signal, so that this could be accounted for during analysis:

*“We have not been doing any additional chemistry on the samplers as we were told we could not. With our other samples, and this will apply to the hospital samples as well, we measure some basic chemistry which we use to normalize the flow but we were told at a very early stage with the prisons wastewater project that we were not to do anything other than measure SARS-CoV-2.”* (Academic Partner)

### Sensitivity and positive predictive value

The sensitivity of a surveillance system indicates its ability to detect a real increase or decrease in the population being surveyed. Conversely, predictive positive value of the system refers to whether a signal identified by the surveillance system accurately represents a real change in the population.

Between 17th May and 29th December 2022, a total of 12 High-level, 30 Rapid Increase and 8 Increasing Trend signals were triggered. These signal triggers generally matched with those trends observed within existing surveillance.

The prison wastewater surveillance system detected trends signaling changes in occurrence of disease, which can be collaborated via traditional surveillance systems (Figure 2). Spikes in cases noted via traditional surveillance methodology were generally followed by similar spikes within the wastewater signal, although there were exceptions. The wave of infections over the summer period between June and August 2022 (weeks 25–35) were captured, with a lag of between 1 and 2 weeks behind existing surveillance and tended to drop off earlier.

Tracking the positive-predictive value of the wastewater surveillance is challenging due to the lack of a ‘gold standard’ with which to compare, with changes in testing policy over the period. However, participants noted that while wastewater generally seems to match what they are already aware of via existing surveillance, sometimes it did not correlate:

“O*ur experience from it when it was first introduced is that sometimes when you saw a high signal, and this was in the time when we still had asymptomatic testing, we have not seen the cases. Equally, we have seen it when it does seem to correspond, so I think we have had a lack of understanding as to what it’s telling us.*” (CCDC)

There were occasions described where either known clusters did not seem to present in the wastewater data, or where fluctuations in SARS-CoV-2 levels in the water did not correlate with existing surveillance, which led to some confusion over how to interpret the data and how much weight to place on the data.

Analysis confirmed that up to 18th July 2022 (week 29), there was some evidence that wastewater surveillance matched case data from existing surveillance. There was a correlation [*r*_(32)_ 0.47, *p* < 0.01] between mean wastewater levels and weekly confirmed cases of SARS-CoV-2. However, as is clear from Figure 2, there was no longer any evidence that the wastewater signal was predictive of case numbers [*r*_(19)_ -0.13, *p* = 0.56] after 18th July 2022. Symptomatic testing was available throughout the study period and asymptomatic testing ceased on the 8th September 2022, although may have been reintroduced in response to local risk and outbreak management (25). This suggests that the divergence of the signals could not be accounted for by changes in testing policy within the prisons over the period.

Of the 328 wastewater samples obtained from the four prisons, 137 did not meet the threshold for detection and were consequently excluded from the analysis (53 samples from Prison D, 35 from Prison B, 33 from Prison C and 16 from Prison A).

### Representativeness

The wastewater surveillance pilot covered four of the six prisons in Wales. Wastewater surveillance was intended for localized action and was representative of those prisons under surveillance but was not intended to provide a signal for the entire Welsh prison estate.

While wastewater surveillance is able to characterize the distribution of COVID-19 infection over time, it lacks the granularity to describe the spread of infection at an individual or hyperlocal level. Due to wastewater capturing sewage from the entire prison, data reflect both residents and staff within the facility. Similarly, the sampling approach meant there was no way to know which area of the prison were responsible for increases in COVID-19 infections, meaning there was no way to target interventions at particular groups or places, limiting the usefulness of the system to implement actions to manage outbreaks.

The samplers were placed in locations that capture the wastewater of the entire prison so is representative of the prison as a whole. However, academic partners had no knowledge of where samplers were located within the prison site. They felt this limited their ability to properly interpret the data:

*“We have no information [about the sampler location], we have asked but its information that will not be shared with us. It would be good to have an outline of the network and where they are, we could then update the report to reflect that.”* (Academic Partner)

CCDC’s and prison governors also had no knowledge of the sampler location, which led to some confusion over whether the data provided a representative sample of the prison or whether particular areas of the prison were biased. As a part of this evaluation, site maps with the sampler locations were acquired from Welsh Government and shared with both CCDC’s and Governors, but not academic partners.

### Timeliness

All participants described receiving the reports in a timely manner from PHW. Reports are distributed weekly, but not always on the same day. There was a lag that ranged between 5 and 7 days between sample collection and report distribution, with median of six. It should be noted, however, that the turnaround time between sample collection and qPCR analysis was rapid (24–48 h) with the lag associated with the decision to only report formally on a weekly basis. The lag was more prominent (3 weeks) for variant surveillance due to lab processes taking longer to complete, with data generation taking 2 weeks and reporting adding an additional week.

## Discussion

### Principal findings

Whilst wastewater surveillance has been important worldwide in providing intelligence on the prevalence of SARS-CoV-2 in the community, we found its utility in the prison setting less clear. In our evaluation, stakeholders found the SARS-CoV-2 wastewater monitoring was a complimentary adjunct to existing surveillance, supporting previous literature (3, 26–28). However, there were several limitations reported that should be addressed to improve its usefulness as a surveillance tool in closed settings during future SARS-CoV-2 waves or other pandemics.

Wastewater had limited usefulness in responding to outbreaks of SARS-CoV-2 within the prison estate. Aligning with other research regarding prison wastewater surveillance systems (28, 29), we found that the inability to identify areas or populations within the prison created frustrations for infection control and limited ability to target interventions. The system was able to describe the distribution of SARS-CoV-2 by time, but not by person or place. In contrast to other literature (3, 17, 27, 28, 30, 31) this near-source study did not support the notion of wastewater providing an early warning system that could support preventative IPC measures. Instead, similarly to a previous qualitative study that considered stakeholder perspectives of wastewater surveillance in prison populations (29), the primary usefulness of prison wastewater surveillance appeared to be in its ability to provide reassurance of non-transmission rather than as a tool to prevent or respond to outbreaks.

As highlighted in a previous review of prison wastewater surveillance (3), it should not replace clinical surveillance for infectious diseases but it can provide useful insight of transmission patterns and can be a useful tool when paired with clinical surveillance data. Similarly to a recent mixed-methods evaluation of a prison wastewater surveillance pilot in Norway, we found that it may represent a more useful surveillance tool during times of reduced testing capacity (32) and help inform decisions regarding when to reintroduce or expand comprehensive clinical testing. Although, our evaluation highlights that targeting these resources remains challenging without an improved sampling strategy.

The novelty of wastewater as a surveillance tool also meant that participants, particularly those without experience in health science, were not confident interpreting the data and had limited or no understanding of the limitations associated with wastewater surveillance. This, coupled with the lack of information sharing between organizations and academic partners, meant no one group had the necessary knowledge to properly interpret the data in the report, calling for improved channels of communication from data collection to data implementation. While those receiving the reports had an understanding of the context on the ground (i.e., population size, prison demographics, access to existing surveillance for cross-checking), they were not as familiar with wastewater surveillance and its interpretation. Conversely, while those supplying the data had the expert knowledge of wastewater surveillance systems, their functionality, strengths and limitations, they had no access to on-the-ground knowledge to properly interpret the data. Skepticism of wastewater variant monitoring expressed by some participants also seemed somewhat unfounded and does not align with published literature (33–35).

Regular feedback is important for well-preforming surveillance systems (36–38). Although academic partners expressed their receptiveness to feedback on the report, no attempts were made to provide or seek feedback by stakeholders besides their participation in this evaluation toward the end of the pilot. For example, visualizations of the data were felt to be the most user-friendly and despite the difficulty interpreting the tabulated data noted by participants, improvements (such as including green and red arrows to indicate positive and negative changes in metrics between reports) to improve clarity and ease of digestion were not suggested but could have been achieved through discussions between stakeholders. Future wastewater projects would benefit from better two-way information sharing with academic partners and a formal feedback process to improve the utility of reports for all stakeholders.

It is also possible that there could have been significant dilution issues in the wastewater caused by rainwater entering the system, dilution from greywater sources or through infiltration of groundwater during times of heavy rain, which could present itself in an inability to detect SARS-CoV-2 even when there were recorded positive cases (Figure 2). Wastewater signals (i.e., SARS-CoV-2 RNA) could also be degraded on some sites because of the samplers proximity to laundry services (3, 39). In other settings, additional chemistry markers such as wastewater ammonium, pH, electrical conductivity and orthophosphate concentrations or biological markers (e.g., crAssphage) are measured and routinely used to account for dilution and disruptive inputs (40). In addition, flow meters can be installed to directly measure wastewater flow. This enables calculation of the total load of SARS-CoV-2 and is far more informative than simply reporting SARS-CoV-2 concentrations in wastewater. However, academic partners in this study were prevented from doing so, which likely negatively impacted the accuracy of wastewater signals. Indeed, the inability for the wastewater surveillance to identify increases in SARS-CoV-2, or specific variants, in specific areas of the prison could have been mitigated by utilizing the expertise of the academic partners when creating the sampling design. Blockages caused by inmates flushing foreign objects can also cause the samplers to fail, necessitating a grab sample on the day which can provide a misleading representation of infection levels.

### Strengths and limitations

A strength of this study is the mixed-methods approach (20), which is rarely used in the evaluation of surveillance systems (41). Qualitative interviews with a breadth of participants gave rich, nuanced detail of how stakeholders with varied priorities within the surveillance system perceived and engaged with wastewater surveillance, painting a detailed picture of the surveillance system, its purpose, intended use, and the way it influences action on the ground. The quantitative element was able to support perspectives provided by participants during the interviews and, to our knowledge, provided the first evidence quantifying the relationship between SARS-CoV-2 levels in wastewater and clinically confirmed cases within a UK prison environment.

While interviews were in-depth, detailed discussions that provided a significant amount of rich, textured data, it is limited by the relatively small number of participants. Secondly, these interviews took place at a time of low COVID-19 prevalence within the prison estate and represent views in the latter stages of the pandemic. Viewpoints may have been different at earlier stages of the pandemic when testing and surveillance infrastructures were less developed. Regarding the quantitative element, changes in clinical testing policy over the study period may have impacted the correlation between the wastewater signal and cases, highlighting a challenge in evaluating the accuracy of wastewater data during periods of fluctuating testing capacity.

Economic evaluation should be a key component in the evaluation of surveillance systems (42) and was included within our interview schedules, however, information on the cost of the wastewater surveillance system was not available to this evaluation. However, previous literature suggests that wastewater-based surveillance offers a cost-effective alternative to traditional surveillance using clinical testing data (43–45).

### Implications for policy and practice

The purpose of surveillance systems are to provide information for action (36). The prison wastewater surveillance program as operated here did not provide information that could, in isolation, lead to public health actions. Instead, it was found to be a useful tool to support existing surveillance. Wastewater surveillance may be more likely to inform public health action where clinical surveillance is unavailable or when a more informative sampling strategy is implemented. Prisons are large, complex environments that require targeted surveillance to inform effective interventions. Future developments to wastewater surveillance that would allow specific areas or populations to be identified, such as samplers in specific blocks of a prison, or faster reporting of sample results, could improve the utility of the wastewater surveillance system to respond to future outbreaks. Improved timeliness of reporting could be achieved via the use of live dashboards to replace weekly reports, reducing the lag between sample collection and report dissemination.

Wastewater surveillance would also benefit a wider sample collection time window than employed here with closer alignment with inmate behavior, alongside involving stakeholders in the design and implementation of future wastewater surveillance programs to improve the usefulness of the system and interpretation of the data provided. Dilution from other water sources such as laundries have been highlighted as challenge for wastewater surveillance programs in prisons (3), and the ability to account for wastewater flow and other sources of water entering the sewerage system (i.e., dilution effects) would also be likely to improve the accuracy of the results obtained. Wastewater has a proven ability in other closed-settings such hospitals (46, 47) and university campuses (48), is adaptable to a range of communicable diseases (49) and represents an important tool in future pandemics within prison settings, particularly if the limitations outlined in this study are addressed.

## Conclusion

In conclusion, wastewater surveillance in closed settings holds value as a complimentary data source to existing case surveillance and represents a non-invasive method of monitoring levels of SARS-CoV-2. In the prison setting, it can detect trends signaling changes in occurrence of SARS-CoV-2 that matched relatively consistently with known cases. However, several important limitations remain that hinder its usefulness in responding to outbreaks and implementing control measures. To strengthen its standing as a useful near-source surveillance system in the future, improvements are needed to the timeliness of reporting, the ability to account for sample dilution, and the ability to narrow findings to localized areas within the prison through strengthened sampling strategies, alongside improved communication and feedback channels between stakeholders.

## Data Availability

The raw data analysed for this article are not readily available because they identify either specific prisons or individuals. Requests to access aggregate and anonymised datasets will be considered and should be directed to gethin. jones6@wales.nhs.uk.

## Footnotes

^1^These posts were created within each prison to provide a single point of contact for COVID-19 related queries.

^2^Academic partners indicated that HMPPS expressed concerns over confidentiality and wanted to limit any additional chemistry, with speculation that this may be to avoid detection of other compounds that could be used to monitor drug use.

## References

[1] Tavoschi L O’Moore É Hedrich D. Challenges and opportunities for the management of infectious diseases in Europes’ prisons: evidence-based guidance. Lancet Infect Dis. (2019) 19:e253–8. doi: 10.1016/S1473-3099(18)30756-4, PMID: 30902441

[2] Burki T. Prisons are “in no way equipped” to deal with COVID-19. Lancet. (2020) 395:1411–2. doi: 10.1016/S0140-6736(20)30984-3, PMID: 32359457 PMC7252088

[3] Hassard F Smith TR Boehm AB Nolan S O’Mara O Di Cesare M. Wastewater surveillance for rapid identification of infectious diseases in prisons. Lancet Microbe. (2022) 3:e556–7. doi: 10.1016/S2666-5247(22)00154-9, PMID: 35688168 PMC9173719

[4] Braithwaite I Edge C Lewer D Hard J. High COVID-19 death rates in prisons in England and Wales, and the need for early vaccination. Lancet Respir Med. (2021) 9:569–70. doi: 10.1016/S2213-2600(21)00137-5, PMID: 33740466 PMC7963444

[5] Gulati G Dunne CP Kelly BD. Prisons and the COVID-19 pandemic. Ir J Psychol Med. (2021) 38:232–3. doi: 10.1017/ipm.2020.65, PMID: 32456716 PMC7294073

[6] Kinner SA Young JT Snow K Southalan L Lopez-Acuña D Ferreira-Borges C. Prisons and custodial settings are part of a comprehensive response to COVID-19. Lancet Public Health. (2020) 5:e188–9. doi: 10.1016/S2468-2667(20)30058-X, PMID: 32197116 PMC7103922

[7] Environmental Modelling Group (EMG) Transmission Group. COVID-19 transmission in prison settings. (2021). Available at: https://www.gov.uk/government/publications/emg-transmission-group-covid-19-transmission-in-prison-settings-25-march-2021 (Accessed October 16, 2022)

[8] Edge C Hard J Wainwright L Gipson D Wainwright V Shaw J. COVID-19 and the prison population (working paper). Health Foundation; (2021). Available at: https://www.health.org.uk/publications/covid-19-and-the-prison-population (Accessed October 28, 2024).

[9] Hawks L Woolhandler S McCormick D. COVID-19 in prisons and jails in the United States. JAMA Intern Med. (2020) 180:1041. doi: 10.1001/jamainternmed.2020.1856 32343355

[10] Perry WB Chrispim MC Barbosa MRF De Souza LM Razzolini MTP Nardocci AC. Cross-continental comparative experiences of wastewater surveillance and a vision for the 21st century. Sci Total Environ. (2024) 919:170842. doi: 10.1016/j.scitotenv.2024.170842 38340868

[11] Jones DL Baluja MQ Graham DW Corbishley A McDonald JE Malham SK. Shedding of SARS-CoV-2 in feces and urine and its potential role in person-to-person transmission and the environment-based spread of COVID-19. Sci Total Environ. (2020) 749:141364. doi: 10.1016/j.scitotenv.2020.141364, PMID: 32836117 PMC7836549

[12] Wang T Wang C Myshkevych Y Mantilla-Calderon D Talley E Hong PY. SARS-CoV-2 wastewater-based epidemiology in an enclosed compound: a 2.5-year survey to identify factors contributing to local community dissemination. Sci Total Environ. (2023) 875:162466. doi: 10.1016/j.scitotenv.2023.162466, PMID: 36868271 PMC9977070

[13] Davó L Seguí R Botija P Beltrán MJ Albert E Torres I. Early detection of SARS-CoV-2 infection cases or outbreaks at nursing homes by targeted wastewater tracking. Clin Microbiol Infect. (2021) 27:1061–3. doi: 10.1016/j.cmi.2021.02.003, PMID: 33601008 PMC7882920

[14] Welsh Government. Wastewater monitoring reports: coronavirus. (2022).Available at: https://www.gov.wales/wastewater-monitoring-reports-coronavirus (Accessed October 28, 2024).

[15] Callahan C Ditelberg S Dutta S Littlehale N Cheng A Kupczewski K. Saliva is comparable to nasopharyngeal swabs for molecular detection of SARS-CoV-2. Powell EA, editor. Microbiol Spectr. (2021) 9:e00162–21. doi: 10.1128/Spectrum.00162-21 PMC8552668 34406838

[16] Schmitz BW Innes GK Prasek SM Betancourt WQ Stark ER Foster AR. Enumerating asymptomatic COVID-19 cases and estimating SARS-CoV-2 fecal shedding rates via wastewater-based epidemiology. Sci Total Environ. (2021) 801:149794. doi: 10.1016/j.scitotenv.2021.149794, PMID: 34467933 PMC8378060

[17] Polo D Quintela-Baluja M Corbishley A Jones DL Singer AC Graham DW. Making waves: wastewater-based epidemiology for COVID-19 – approaches and challenges for surveillance and prediction. Water Res. (2020) 186:116404. doi: 10.1016/j.watres.2020.116404, PMID: 32942178 PMC7480445

[18] European Centre for Disease Prevention and Control. Data quality monitoring and surveillance system evaluation: a handbook of methods and applications. Eur Centre Disease Prevent Control. (2014). doi: 10.2900/35329

[19] German RR Lee LM Horan JM Milstein RL Pertowski CA Waller MN. Updated guidelines for evaluating public health surveillance systems: recommendations from the guidelines working group. MMWR Recomm Rep Morb Mortal Wkly Rep Recomm Rep. (2001) 50:1–35. 18634202

[20] Wasti SP Simkhada P Van Teijlingen E Sathian B Banerjee I. The growing importance of mixed-methods research in health. Nepal J Epidemiol. (2022) 12:1175–8. doi: 10.3126/nje.v12i1.43633 35528457 PMC9057171

[21] Kiger ME Varpio L. Thematic analysis of qualitative data: AMEE guide no. 131. Med Teach. (2020) 42:846–54. doi: 10.1080/0142159X.2020.1755030, PMID: 32356468

[22] Keck JW Lindner J Liversedge M Mijatovic B Olsson C Strike W. Wastewater surveillance for SARS-CoV-2 at long-term care facilities: mixed methods evaluation. JMIR Public Health Surveill. (2023) 9:e44657. doi: 10.2196/44657, PMID: 37643001 PMC10467632

[23] Farkas K Hillary LS Thorpe J Walker DI Lowther JA McDonald JE. Concentration and quantification of SARS-CoV-2 RNA in wastewater using polyethylene glycol-based concentration and qRT-PCR. Methods Protoc. (2021) 4:17. doi: 10.3390/mps4010017, PMID: 33672247 PMC8005995

[24] R Core Team. R: A language and environment for statistical computing [internet]. Vienna, Austria; (2023). Available at: https://www.R-project.org/ (Accessed October 28, 2024).

[25] Ministry of Justice. HM prison and probation service COVID-19 official statistics, November 2022. Ministry of Justice. (2022). Available at: https://www.gov.uk/government/statistics/hmpps-covid-19-statistics-november-2022/hm-prison-and-probation-service-covid-19-statistics-november-2022 (Accessed October 28, 2024).

[26] Hrudey SE Conant B. The devil is in the details: emerging insights on the relevance of wastewater surveillance for SARS-CoV-2 to public health. J Water Health. (2022) 20:246–70. doi: 10.2166/wh.2021.186, PMID: 35100171

[27] Zhang D Duran SSF Lim WYS Tan CKI Cheong WCD Suwardi A. SARS-CoV-2 in wastewater: from detection to evaluation. Mater Today Adv. (2022) 13:100211. doi: 10.1016/j.mtadv.2022.100211, PMID: 35098102 PMC8786653

[28] Klevens RM Young CCW Olesen SW Osinski A Church D Muten J. Evaluation of wastewater surveillance for SARS-CoV-2 in Massachusetts correctional facilities, 2020–2022. Front Water. (2023) 5:1083316. doi: 10.3389/frwa.2023.1083316

[29] Harris-Lovett S Nelson KL Kantor R Korfmacher KS. Wastewater surveillance to inform public health decision making in residential institutions. J Public Health Manag Pract. (2023) 29:317–21. doi: 10.1097/PHH.0000000000001636, PMID: 36214654 PMC10038809

[30] Bogler A Packman A Furman A Gross A Kushmaro A Ronen A. Rethinking wastewater risks and monitoring in light of the COVID-19 pandemic. Nat Sustain. (2020) 3:981–90. doi: 10.1038/s41893-020-00605-2

[31] Kapoor V Al-Duroobi H Phan DC Palekar RS Blount B Rambhia KJ. Wastewater surveillance for SARS-CoV-2 to support return to campus: methodological considerations and data interpretation. Curr Opin Environ Sci Health. (2022) 27:100362. doi: 10.1016/j.coesh.2022.100362, PMID: 35402756 PMC8975751

[32] Amato E Hyllestad S Heradstveit P Langlete P Moen LV Rohringer A. Evaluation of the pilot wastewater surveillance for SARS-CoV-2 in Norway, June 2022 – march 2023. BMC Public Health. (2023) 23:1714. doi: 10.1186/s12889-023-16627-2, PMID: 37667223 PMC10476384

[33] Bar-Or I Weil M Indenbaum V Bucris E Bar-Ilan D Elul M. Detection of SARS-CoV-2 variants by genomic analysis of wastewater samples in Israel. Sci Total Environ. (2021) 789:148002. doi: 10.1016/j.scitotenv.2021.148002, PMID: 34323811 PMC8142738

[34] Karthikeyan S Levy JI De Hoff P Humphrey G Birmingham A Jepsen K. Wastewater sequencing reveals early cryptic SARS-CoV-2 variant transmission. Nature. (2022) 609:101–8. doi: 10.1038/s41586-022-05049-6, PMID: 35798029 PMC9433318

[35] Xu X Deng Y Ding J Zheng X Wang C Wang D. Wastewater genomic sequencing for SARS-CoV-2 variants surveillance in wastewater-based epidemiology applications. Water Res. (2023) 244:120444. doi: 10.1016/j.watres.2023.120444, PMID: 37579567

[36] Groseclose SL Buckeridge DL. Public health surveillance systems: recent advances in their use and evaluation. Annu Rev Public Health. (2017) 38:57–79. doi: 10.1146/annurev-publhealth-031816-044348 27992726

[37] Peyre M Hoinville L Njoroge J Cameron A Traon D Goutard F. The RISKSUR EVA tool (Survtool): a tool for the integrated evaluation of animal health surveillance systems. Prev Vet Med. (2019) 173:104777. doi: 10.1016/j.prevetmed.2019.104777, PMID: 31731037

[38] Alemu T Gutema H Legesse S Nigussie T Yenew Y Gashe K. Evaluation of public health surveillance system performance in Dangila district, Northwest Ethiopia: a concurrent embedded mixed quantitative/qualitative facility-based cross-sectional study. BMC Public Health. (2019) 19:1343. doi: 10.1186/s12889-019-7724-y, PMID: 31640662 PMC6805593

[39] Silverman AI Boehm AB. Systematic review and Meta-analysis of the persistence of enveloped viruses in environmental waters and wastewater in the absence of disinfectants. Environ Sci Technol. (2021) 55:14480–93. doi: 10.1021/acs.est.1c03977 34665598

[40] Wilde H Perry WB Jones O Kille P Weightman A Jones DL. Accounting for dilution of SARS-CoV-2 in wastewater samples using Physico-chemical markers. Water. (2022) 14:2885. doi: 10.3390/w14182885

[41] Drewe JA Hoinville LJ Cook AJC Floyd T Stärk KDC. Evaluation of animal and public health surveillance systems: a systematic review. Epidemiol Infect. (2012) 140:575–90. doi: 10.1017/S0950268811002160, PMID: 22074638

[42] Calba C Goutard FL Hoinville L Hendrikx P Lindberg A Saegerman C. Surveillance systems evaluation: a systematic review of the existing approaches. BMC Public Health. (2015) 15:448. doi: 10.1186/s12889-015-1791-5, PMID: 25928645 PMC4418053

[43] Ngwira LG Sharma B Shrestha KB Dahal S Tuladhar R Manthalu G. Cost of wastewater-based environmental surveillance for SARS-CoV-2: evidence from pilot sites in Blantyre, Malawi and Kathmandu, Nepal. PLOS Glob Public Health. (2022) 2:e0001377. doi: 10.1371/journal.pgph.0001377 36962924 PMC10021894

[44] Wright J Driver EM Bowes DA Johnston B Halden RU. Comparison of high-frequency in-pipe SARS-CoV-2 wastewater-based surveillance to concurrent COVID-19 random clinical testing on a public U.S. university campus. Sci Total Environ. (2022) 820:152877. doi: 10.1016/j.scitotenv.2021.152877, PMID: 34998780 PMC8732902

[45] Yoo BK Iwamoto R Chung U Sasaki T Kitajima M. Economic evaluation of wastewater surveillance combined with clinical COVID-19 screening tests, Japan. Emerg Infect Dis. (2023) 29:1608–1617. doi: 10.3201/eid2908.221775 37486197 PMC10370838

[46] Acosta NA Bautista M Hollman J Mccalder J Beaudet AB Man L. Wastewater monitoring of SARS-CoV-2 from acute care hospitals identifies nosocomial transmission and outbreaks. Infectious Diseases. (2021). doi: 10.1101/2021.02.20.21251520

[47] Acosta N Bautista MA Hollman J McCalder J Beaudet AB Man L. A multicenter study investigating SARS-CoV-2 in tertiary-care hospital wastewater. Viral burden correlates with increasing hospitalized cases as well as hospital-associated transmissions and outbreaks. Water Res. (2021) 201:117369. doi: 10.1016/j.watres.2021.117369, PMID: 34229222 PMC8214445

[48] Scott LC Aubee A Babahaji L Vigil K Tims S Aw TG. Targeted wastewater surveillance of SARS-CoV-2 on a university campus for COVID-19 outbreak detection and mitigation. Environ Res. (2021) 200:111374. doi: 10.1016/j.envres.2021.111374, PMID: 34058182 PMC8163699

[49] Kilaru P Hill D Anderson K Collins MB Green H Kmush BL. Wastewater surveillance for infectious disease: a systematic review. Am J Epidemiol. (2023) 192:305–22. doi: 10.1093/aje/kwac175, PMID: 36227259 PMC9620728

