# Supplementary figures and images for: Evaluation of wastewater surveillance for SARS-CoV-2 in a prison population: a mixed-methods approach
Source: Front Public Health. 2024 Nov 19;12:1462186. doi: 10.3389/fpubh.2024.1462186 (PMC11611585; doi:10.3389/fpubh.2024.1462186)

## Supplement 1: Example of Thematic Analysis of Qualitative Interviews

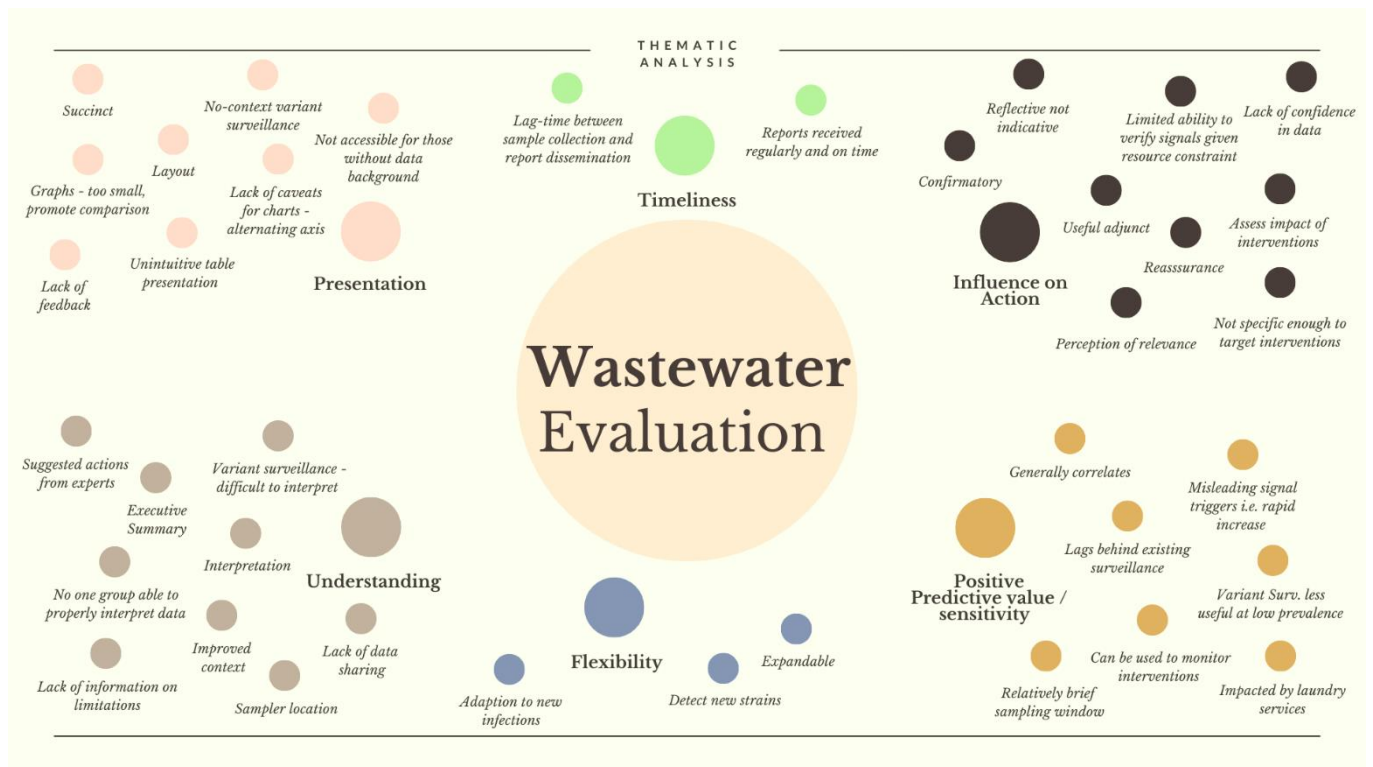

Supplement: Supplementary file 1 [file Data_Sheet_1.PDF]
