# Supplementary material for: Evaluation of wastewater surveillance for SARS-CoV-2 in a prison population: a mixed-methods approach
Source: Front Public Health. 2024 Nov 19;12:1462186. doi: 10.3389/fpubh.2024.1462186 (PMC11611585; doi:10.3389/fpubh.2024.1462186)
Supplement: Supplementary file 2 [file Data_Sheet_2.PDF]

Supplement 2: Prison Wastewater Surveillance Interview Schedules

Interview schedule: those receiving reports

Introduction

- Introductions
- Discuss purpose of evaluation and what will be covered during the interview
    - Would like to examine the use of wastewater surveillance within prisons in Wales to consider if it contributes to the prevention and control of adverse effects from COVID-19, including how it is used by stakeholders like yourself and what actions, if any, are implemented following the data in the report
    - We'll be looking at several attributes during the evaluation, but the ones I'd like to focus on in this interview are timeliness, usefulness and acceptability
- Discuss confidentiality and anonymity
- Discuss recording of interview, how it will be used and after what time it will be destroyed
- Semi-structured interview so while we have a schedule to follow, there is room to digress and follow conversation naturally

Timeliness

- Do you receive the wastewater reports completed by Welsh Government?
    - Do you receive them in a regular and timely manner?

Usefulness

- Do you understand the data in the reports?
    - Do you understand how to interpret the data?
- Do you understand the limitations of the data in the reports?
- How useful do you find the wastewater reports?
    - Which elements of the report do you find most beneficial?
- Have the reports influenced outbreak investigations on site?
    - If so, how?
- What actions, if any, have you implemented based on the data in these reports?
    - If you are informed that one of the signals has been triggered (high-level/rapid/increasing trend), are any actions taken as a result?
- Do you find the information on variant surveillance useful?

Acceptability

- Do you feel like you have an avenue or opportunity to provide feedback on the report?
- Is there anything in the report you would like to see improved?
- Has the implementation of wastewater surveillance placed any additional burden on you in terms of time and resources?

Interview Schedule: those involved in producing reports

Introduction

- Introductions
- Discuss purpose of evaluation and what will be covered during the interview
  - Would like to examine the use of wastewater surveillance within prisons in Wales to consider if it contributes to the prevention and control of adverse effects from COVID-19
  - We'll be looking at several attributes during the evaluation, but the ones I'd like to focus on in this interview are usefulness, flexibility, data quality, and representativeness, cost and resources and lessons learned
- Discuss confidentiality and anonymity
- Discuss recording of interview, how it will be used and after what time it will be destroyed
- Semi-structured interview so while we have a schedule to follow, there is room to digress and follow conversation naturally

Usefulness:

- How should you interpret the data in the reports?
- What are the limitations of the reports?
  - What conclusions are you able to draw from the report and what limitations are associated?
- How should the data included in the report be acted upon?
  - What predictive value do they have?

Flexibility:

- Is the wastewater surveillance system able to detect unknown variants?
  - Are these new variants able to be studied, or just reported on?
- Could the current system be adapted to allow for surveillance of other infections such as influenza or norovirus?
- How easily could additional prisons be added to the system?

Data quality:

- What factors may influence insufficient/poor quality samples?

Representativeness:

- Do the samples taken provide a representative sample of the prison?
- Does the placement of the sampler bias wings/blocks that are situated closer to where the samples are taken?

Cost/resources:

- How much did the system cost to set-up, and how much does it cost to maintain?
- How much do the samplers cost, and how much do they cost to install and maintain?
- How much resource does the system require to run in terms of time and staff?

Community wastewater surveillance

- Has the prison wastewater surveillance programme contribute to improved understanding of community wastewater surveillance? Does one compliment the other? Are drawing inferences easier with community or prison wastewater surveillance?

Lessons learned

- What lessons have you learned from the wastewater surveillance programme?
