# Supplementary material for: Evaluation of wastewater surveillance for SARS-CoV-2 in a prison population: a mixed-methods approach
Source: Front Public Health. 2024 Nov 19;12:1462186. doi: 10.3389/fpubh.2024.1462186 (PMC11611585; doi:10.3389/fpubh.2024.1462186)

HMPS Wastewater Monitoring Report: 6 December 2022

Samples taken up to: 30 November 2022

Wastewater-based epidemiology provides comprehensive public health information at a community level. Individuals with COVID-19 shed SARS-CoV-2 genetic material in their faeces in the form of ribonucleic acid (RNA), regardless of whether they have symptoms. This information provides a representative and unbiased snapshot of the level of COVID-19 infection within the surveyed community at any point in time.

The key data reported in this report are the load of SARS-CoV-2 viral gene copies in the wastewater samples. Currently, a mixture of ‘composite’ and ‘spot’ one litre samples are collected from each of the four prison sites, three times a week. Spot samples are taken at the same time each day, while composite samples are collected over a 3-hour period using automatic sampling machines. In a week where a mixture of composite and spot samples are used, this is indicated in the below ‘Sampling type’ column as ‘mixed’. Details on the SARS-CoV-2 quantification process can be found in any Welsh Government Wastewater Monitoring in Wales Report. Adverse noise in the SARS-CoV-2 load is smoothed using a 10-day rolling average, and the data are scaled by 10^4 to improve readability. Variants are identified using next generation sequencing of the SARS-CoV-2 genome, and are presented as their relative abundances in the sample.

To highlight potentially concerning changes in wastewater signal, three indicators are assessed once a week, based on the end-of-week, smoothed SARS-CoV-2 load. These indicators are presented in the indicator table below and are defined as follows:

1. The **High Signal Level** indicator highlights the sites where viral load is high. It corresponds to a situation where the viral load exceed half of the highest recorded load at the site in the last six months.
2. The **Rapid Increase** indicator highlights the sites where viral load is increasing at an alarming rate. It corresponds to a situation where the end-of-week load has increased by at least 100% since the previous week.
3. The **Increasing Signal Level** indicator highlights the sites where viral load is showing signs of continuous increase. It corresponds to a situation where the end-of-week viral load has increased by at least 10% for the last three weeks.

For each indicator, ‘1’ indicates that the indicator triggered at the corresponding site, and ‘0’ means that it did not trigger .Meanwhile, ‘-1’ indicates that there is no (or insufficient) data for that site. Limit of Detection (LOD) is defined as the minimu mconcentration whereby 10 replicates all return positive results. Any questions about this report, or the Welsh Governm entWastewater Programme, can be sent to .

Wastewater signal indicators

Site	Date	End-of-week SARS-CoV-2 level	Successful samples	Samples below LOD	Anomalous samples	Missing sam- ples	High Signal Level	Rapid In- crease	Increasing Signal Level	Sampling type
	04 Dec 2022	24.87	3	0	0	0	0	0	0	Composite
	04 Dec 2022	2.55	1	2	0	0	0	-1	-1	Composite
	04 Dec 2022	18.07	3	0	0	0	0	1	-1	Composite
	04 Dec 2022	0.01	1	2	0	0	0	-1	-1	Composite

Wastewater sequencing results

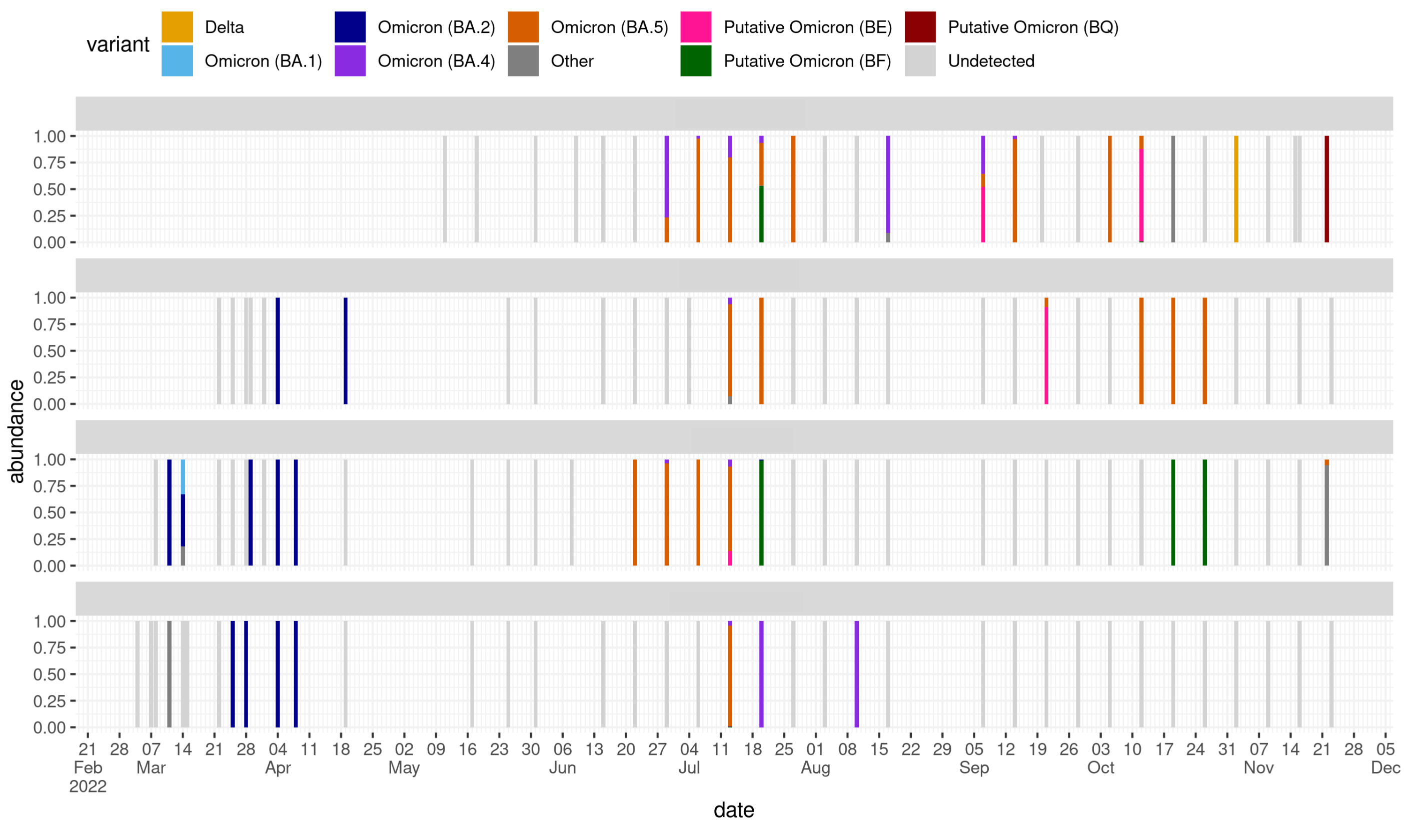

Site-level signal trends

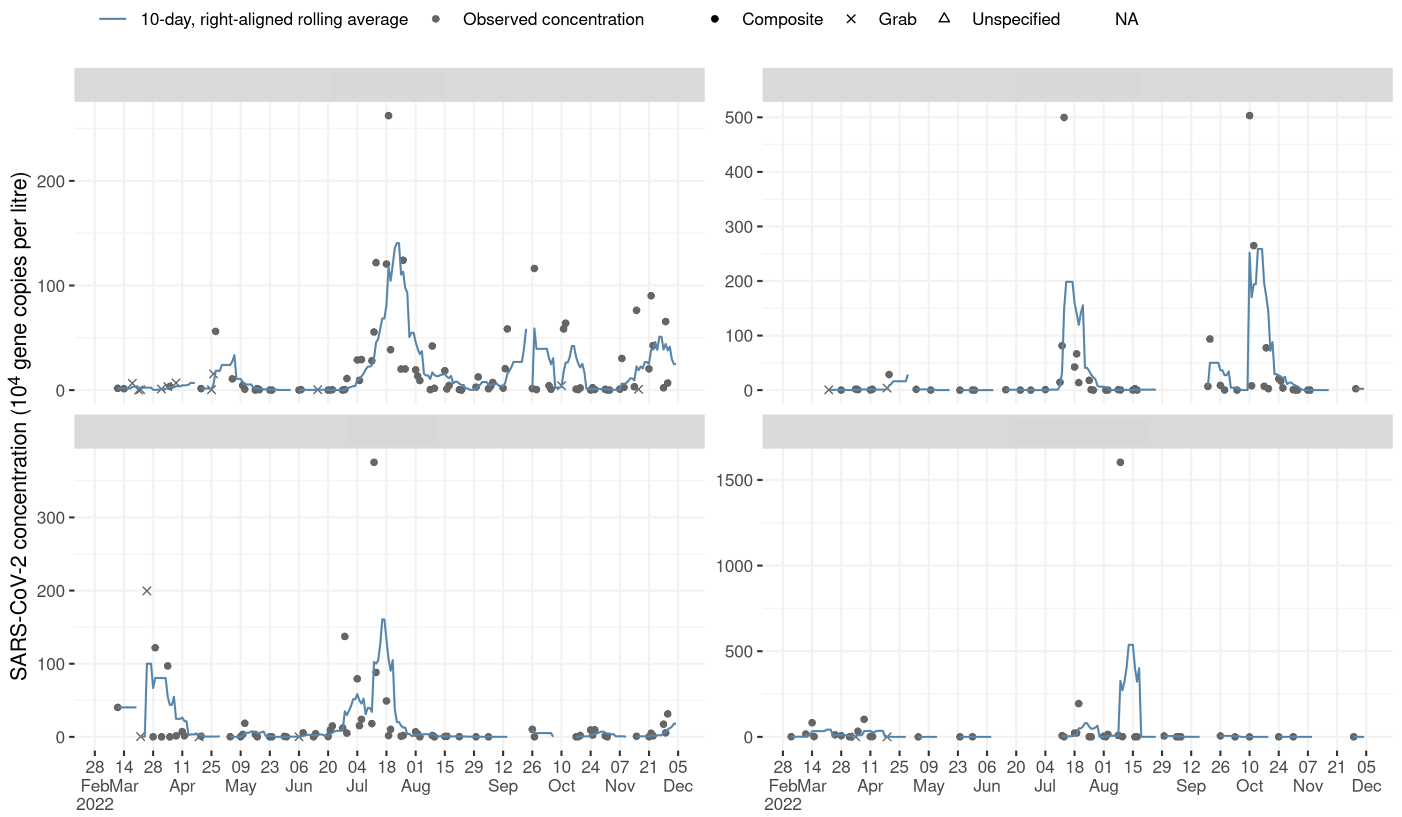

Supplement: Supplementary file 3 [file Data_Sheet_3.pdf]
