# Supplementary material for: Evaluation of wastewater surveillance for SARS-CoV-2 in a prison population: a mixed-methods approach
Source: Front Public Health. 2024 Nov 19;12:1462186. doi: 10.3389/fpubh.2024.1462186 (PMC11611585; doi:10.3389/fpubh.2024.1462186)
Supplement: Supplementary file 4 [file Data_Sheet_4.PDF]

Supplement 4: Amended Version of Table 1 of the Prison Wastewater Surveillance Report produced by HMPPS

	Number of Positive Cases as of 18/10/2022		12-Oct-22				05-Oct-22			
	Prisoners	Staff	End of Week SARS-CoV-2 level	High Level Signal	Rapid Increase Signal	Increasing Trend Signal	End of Week SARS-CoV-2 level	High Level Signal	Rapid Increase Signal	Increasing Trend Signal
**Prison A**	0	8	42.22 ↑	-	✓	-	2.56 ↓	-	-	-
**Prison B**	*	7	258.70 ↑	✓	✓	-	0.04 ↓	-	-	-
**Prison C**	0	6	Samples below LOD				Samples below LOD			
**Prison D**	0	11	0.05 ↓	-	-	-	0.11 ↓	-	-	-

	28-Sep-22				21-Sep-22				14-Sep-22			
	End of Week SARS-CoV-2 level	High Level Signal	Rapid Increase Signal	Increasing Trend Signal	End of Week SARS-CoV-2 level	High Level Signal	Rapid Increase Signal	Increasing Trend Signal	End of Week SARS-CoV-2 level	High Level Signal	Rapid Increase Signal	Increasing Trend Signal
**Prison A**	39.47 ↑	-	-	-	1x below LOD, 1x unsuccessful sample				27.0 ↑	-	✓	-
**Prison B**	4.56 ↓	-	-	-	50.19 ↑	-	-	-	Samples below LOD			
**Prison C**	5.19 ↑	-	-	-	Samples below LOD				Samples below LOD			
**Prison D**	5.69 ↑	-	-	-	Samples below LOD				Samples below LOD			
