# Supplementary material for: Evaluation of wastewater surveillance for SARS-CoV-2 in a prison population: a mixed-methods approach
Source: Front Public Health. 2024 Nov 19;12:1462186. doi: 10.3389/fpubh.2024.1462186 (PMC11611585; doi:10.3389/fpubh.2024.1462186)
Supplement: Supplementary file 5 [file Data_Sheet_5.PDF]

Supplement 5: Evaluation attribute definitions and summary of findings

Surveillance attribute	Strengths	Limitations
**Usefulness** refers to how well a system preforms in terms of attributable public health action (ECDC). We examine whether wastewater surveillance was a useful tool in responding to possible outbreaks and what actions resulted from the data provided, alongside to what extent stakeholders understood the data provided.	+ Wastewater surveillance was a useful adjunct to support existing clinical surveillance, providing important intelligence on the levels of SARS-CoV-2 circulating within the prison<br>+ Provided reassurance of decreased transmission and efficacy of interventions, particularly during times of reduced testing capacity<br>+ Contributed to decisions to close-down outbreak investigations	– Did not provide information that could, in isolation, lead to useful public health action<br>– Wastewater was not specific enough to target interventions (i.e. through inability to specify locations/populations associated with increased SARS-CoV-2 levels)<br>– Wastewater did not provide the leading indicator expected and instead lagged behind existing clinical surveillance, limiting its usefulness in responding to signals<br>– Limited understanding of how to interpret the data included within reports among some stakeholders without scientific backgrounds<br>– Lack of information sharing between organisations and academic partners involved meant no one group had the necessary knowledge to properly interpret the data<br>– Limited perceived usefulness of variant surveillance, particularly given the lack of interpretation provided
**Flexibility** refers to the ability of a surveillance system to adapt to changing information or operational needs (CDC). We examine whether the system could adapt to alternate pathogens and variants, alongside how easily new sites could be added to the system.	+ Able to detect both existing and emerging SARS-CoV-2 variants<br>+ Able to adapt to alternate infections such as influenza or norovirus<br>+ New sites could be added with relative ease, the only barrier being the cost and logistics and sample collection and analysis	
**Acceptability** refers to the willingness of stakeholders to engage with the surveillance system (ECDC). We examine whether the introduction of wastewater surveillance placed any additional burden on stakeholders and their perspectives on how the data was presented in reports.	+ Introduction of wastewater surveillance programme placed no additional burden in terms of time or resources on stakeholders<br>+ Visual interpretation of data was preferred<br>+ Brevity and concise nature of report commended<br>+ Good engagement with report despite reservations surrounding appropriate interpretation	– No formal feedback mechanism implemented<br>– Signal triggers table felt to be unintuitive and confusing, with the numbering system difficult to understand<br>– Rapid Increase signal trigger potentially misleading given its reliance on the previous weeks data (i.e. doubling of a low and high viral load would both represent a 100% increase and trigger the Rapid Increase indicator)
**Data quality** refers to the completeness and validity of the data collected and reported by the surveillance system (ECDC). We examine the completeness of data provided and what factors impact data quality of samples.	+ Only a small proportion of samples were missed across all participating sites<br>+ Missing samples did not occur with any regularity but were often aggregated	– Data quality negatively impacted by 'ragging' (i.e. blockages caused by foreign objects flushed down toilets) and the samplers' proximity to laundry services in some prisons, limiting the validity of results. The latter can be accounted for in analysis, but academic partners were prevented from doing any additional chemistry on the samples.<br>– The necessity of 'grab samples' when composite autosamplers fail can introduce bias to results
**Sensitivity** was defined as the ability of the surveillance system to detect true signal changes over time (CDC). This was evaluated by counting the number of signal trends triggered throughout the period and examining whether these correlated with on-site intelligence	+ Between 17th May and 29th December 2022, a total of 12 High-level, 30 Rapid Increase and 8 Increasing Trend signals were triggered, with signal triggers generally matched with trends observed within existing surveillance	– Qualitative evidence suggests signal triggers did not always correlate with intelligence on the ground

**Positive predictive value** was defined as whether or not signals identified by the surveillance system accurately represent real changes in the population. We examined this by investigating whether trends observed via wastewater surveillance reflect what was observed through clinical surveillance.	+ Trends identified by wastewater surveillance signalling changes in occurrence of disease were collaborated via traditional surveillance systems, with spikes in cases identified via clinical surveillance generally followed by similar spikes within the wastewater signal<br>+ The wave of infections over the summer period between June and August 2022 (weeks 25-35) were captured, with a lag of between one and two weeks behind existing surveillance<br>+ Correlation observed between mean wastewater levels of weekly confirmed SARS-CoV-2 cases ($r_{(32)}$ 0.47, $p<0.01$)	– No evidence that wastewater signal was predictive of case numbers after 18th July 2022 ($r_{(19)}$ -0.13, p = 0.56), which cannot be accounted for by changes in testing policy<br>– Qualitative evidence suggests occasions where either know clusters did not seem to present in wastewater data, or where fluctuations in wastewater signals did not correlate with clinical surveillance
**Representativeness** refers to the ability of a surveillance system to accurately describe the distribution of infection within the population by place and person (CDC)	+ Captured the entirety of the prison population in those prisons included in the wastewater surveillance pilot and therefore provided a representative picture of the prison as a whole<br>+ Able to characterise the distribution of SARS-CoV-2 over time in each respective prison	– As sampling captured sewage from the entire prison, data reflects both staff and prisons, with no ability to distinguish between population groups<br>– Lacks granularity to describe data at a hyperlocal level, limiting usefulness to identify particular areas of the prison responsible for increased levels of infection<br>– Sampler locations were not shared with academic partners, hindering their ability to properly interpret the data<br>– Only 4 of 6 prisons were included in the wastewater surveillance pilot, meaning it could not provide a signal for the entire Welsh prison estate<br>– Limited sampling window (7am-10am) and occasional reliance on 'grab samples' (taken at time of collection if sampler failed) meant data is not a true reflection of the levels of SARS-CoV-2 throughout the day
**Timeliness** describes the speed between steps in a surveillance system (CDC)	+ Reports were distributed weekly in a timely and consistent manner<br>+ Turnaround time of 24-24 hours possible	– Despite the possibility of a quicker turnaround time, decision was taken to only report weekly<br>– Median lag of 6 days between sample collection and report distribution (range: 5-7 days)<br>– More prominent lag (three-weeks) for variant surveillance due to lab processes taking longer to complete<br>– Reporting lag limited usefulness of information
